# Facial Adiposity, Attractiveness, and Health: A Review

**DOI:** 10.3389/fpsyg.2018.02562

**Published:** 2018-12-21

**Authors:** Stefan de Jager, Nicoleen Coetzee, Vinet Coetzee

**Affiliations:** ^1^Department of Psychology, Sefako Makgatho Health Sciences University, Ga-Rankuwa, South Africa; ^2^Department of Psychology, University of Pretoria, Pretoria, South Africa; ^3^Department of Genetics, Biochemistry and Microbiology, University of Pretoria, Pretoria, South Africa

**Keywords:** facial adiposity, attractiveness, perceived health, health outcomes, BMI, percentage body fat, meta-analysis

## Abstract

The relationship between facial cues and perceptions of health and attractiveness in others plays an influential role in our social interactions and mating behaviors. Several facial cues have historically been investigated in this regard, with facial adiposity being the newest addition. Evidence is mounting that a robust link exists between facial adiposity and attractiveness, as well as perceived health. Facial adiposity has also been linked to various health outcomes such as cardiovascular disease, respiratory disease, blood pressure, immune function, diabetes, arthritis, oxidative stress, hormones, and mental health. Though recent advances in the analysis of facial morphology has led to significant strides in the description and quantification of facial cues, it is becoming increasingly clear that there is a great deal of nuance in the way that humans use and integrate facial cues to form coherent social or health judgments of others. This paper serves as a review of the current literature on the relationship between facial adiposity, attractiveness, and health. A key component in utilizing facial adiposity as a cue to health and attractiveness perceptions is that people need to be able to estimate body mass from facial cues. To estimate the strength of the relationship between perceived facial adiposity and body mass, a meta-analysis was conducted on studies that quantified the relationship between perceived facial adiposity and BMI/percentage body fat. Summary effect size estimates indicate that participants could reliably estimate BMI from facial cues alone (*r* = 0.71, *n* = 458).

## Introduction

Facial appearance in humans conveys a substantial amount of non-verbal information when it comes to our interactions with others. These include judgments of health (Rhodes et al., 2007; Coetzee et al., 2009; Stephen et al., 2009; Phalane et al., 2017), attractiveness (Rhodes, 2006; Coetzee et al., 2012; Foo et al., 2017), leadership ability (Little et al., 2007; Re and Perrett, 2014) and even academic ability (Zebrowitz et al., 2002; Talamas et al., 2016), to name but a few. Naturally, the evolutionary, social and behavioral implications of the way in which we perceive and react to other people's faces, has generated significant interested in the scientific community. Several facial cues have been identified as integral aspects of our judgments of others, including sexual dimorphism (Perrett et al., 1998; Thornhill and Gangestad, 2006), symmetry (Perrett et al., 1999; Scheib et al., 1999; Jones et al., 2001), averageness (Grammer and Thornhill, 1994; Rhodes et al., 2001), skin condition (Jones et al., 2004; Stephen et al., 2011), and facial adiposity, or perceived weight in the face (Coetzee et al., 2009; Tinlin et al., 2013). Of particular interest to scientists is the relationship between facial cues and perceptions of health and attractiveness in others, as these play a particularly influential role in our social interactions and mating behaviors. Since mate choice plays a central role in evolutionary psychology, researchers are eager to understand the relative contribution that various facial cues make in shaping our perceptions and judgments of health and attractiveness (Rhodes, 2006).

Attractiveness plays a prominent role in our everyday interactions with others and research has consistently shown that attractive people are judged more positively in general compared to unattractive people (see Rhodes, 2006; Little et al., 2011). Attractive people are also typically judged to be healthier compared to unattractive people (Kalick et al., 1998; Shackelford and Larsen, 1999; Boothroyd et al., 2013). One potential reason for this is that facial cues associated with attractiveness could also be linked to various health markers. To this end, humans evolved to be particularly attentive to facial cues associated with attractiveness, as these cues allow us to make inferences about other people's health and genetic fitness, or even behavioral tendencies (Rhodes et al., 2001). For example, fitness-related theories of human behavior suggest that key phenotypic cues influence our judgments of others because they evolved as cues to general health and mate quality (Langlois et al., 2000). One example of such a fitness-related theory is the “good genes” hypothesis, which postulates that female mate choice is heavily influenced by phenotypic cues, since they aid females in making snap judgments about men's current health, genetic quality and fertility, and thus suitability for mating (Hamilton and Zuk, 1982; Thornhill and Gangestad, 1993, 1999).

Choosing the right mate could provide substantial direct and indirect benefits to females and their offspring. For example, according to the immunocompetence handicap hypothesis (Folstad and Karter, 1992) males that display strong secondary male sexual traits are perceived as more attractive by females, as these secondary sexual traits may potentially serve as good indicators of immune function in males. The reason for this is that the testosterone that is responsible for the development of these secondary male sexual traits also has an immunosuppressive effect, and only good quality males can therefore afford to display them. By choosing a male with a strong immune system as a mating partner, a female can simultaneously lessen the risk of pathogen exposure, while also maximizing the robustness of her offspring in terms of immune function. It should be mentioned, however, that some studies have found very little to no evidence for a relationship between testosterone and immune suppression in mammals and humans, respectively, thus casting at least some doubt on the viability of the immunocompetence handicap hypothesis (Roberts et al., 2004; Nowak et al., 2018).

From an evolutionary point of view, preferences for certain facial cues as reliable indicators of health and genetic quality, can only be a coherent hypothesis if two conditions are met: (i) there should be agreement between people regarding the facial features that they find attractive (Langlois et al., 2000; Rhodes, 2006) and (ii) perceptions of attractiveness and health should be related to actual health outcomes or genetic quality (Coetzee et al., 2009; Rantala et al., 2013a). At present there is at least some empirical support for the notion that people can reliably detect and agree on facial cues that contribute to facial attractiveness and perceptions of health (Perrett et al., 1998; Langlois et al., 2000; Rhodes et al., 2001; Re and Rule, 2016a). This statement does come with a caveat though, as numerous studies have demonstrated that preferences for certain facial cues, such as facial adiposity for example, are likely mediated by environmental and cultural factors, including resource scarcity (Batres and Perrett, 2014, 2017), exposure to media beauty ideals (Batres and Perrett, 2014), and own ethnicity familiarity (Coetzee et al., 2014; Batres et al., 2017).

While there is strong support for the link between various facial cues and attractiveness, the link between facial attractiveness and actual health outcomes has been mixed (Weeden and Sabini, 2005; Rhodes et al., 2007; Foo et al., 2017; Cai et al., in press). For example, Kalick et al. (1998) found no reliable relationship between rated facial attractiveness and general health rating by physicians for people in adolescence, middle adulthood, or late adulthood. The researchers did, however, find that there was a correlation between rated facial attractiveness and perceived health for both males and females. After controlling for the mediating relationship that attractiveness may play between perceived health and actual health outcomes, a statistically significant correlation between perceived health and actual health was found. The researchers conclude that, paradoxically, attractiveness can cause a halo effect and serve to suppress accurate judgments of health, instead of enhancing them. In partial contrast to Kalick et al. (1998), Henderson and Anglin (2003) did find a significant correlation between rated attractiveness and longevity for 50 facial photographs taken from a high school yearbook, although it is not entirely clear how comparable longevity (Henderson and Anglin, 2003) and general health measures (Kalick et al., 1998) are within this context. For a review on the relationship between attractiveness and health see Weeden and Sabini (2005) and Rhodes (2006).

The relationship between individual facial cues associated with attractiveness and actual health outcomes has also been met with mixed results. For example, a study by Rhodes et al. (2001), using a very similar dataset to Kalick et al. (1998), found a relationship between facial averageness and perceived health, as well as facial symmetry and perceived health for both males and females. However, when the researchers investigated the potential relationship between actual health outcomes and facial averageness, a correlation was found only for male childhood health (*r* = 0.28), female adolescent health (*r* = 0.14) and the current health for females (*r* = 0.25). For rated asymmetry, no link was found between health outcomes and perceived symmetry of the participant's faces. One potential explanation for this inconsistent trend is that general health is a very broad concept that probably reflects a wide variety of health markers, which may, or may not, be linked to particular facial cues or even general facial attractiveness.

### Facial Adiposity as a Cue to Perceived Health and Attractiveness

One of the facial cues that has consistently been associated with both attractiveness (Coetzee, 2011; Coetzee et al., 2012; Rantala et al., 2013a; Foo et al., 2017; Phalane et al., 2017), perceived health (Coetzee et al., 2009; Fisher et al., 2014b; Han et al., 2016; Windhager et al., 2018) and actual health outcomes (Coetzee et al., 2009; Reither et al., 2009; Rantala et al., 2013a; Tinlin et al., 2013; Martinson and Vasunilashorn, 2016) is facial adiposity. Facial adiposity, or perceived weight in the face, was demonstrated to be a reliable cue to health in a study published by Coetzee et al. (2009) where higher facial adiposity ratings were linked to perceived health and attractiveness, as well as increased risk of infection and cardiovascular-illness. Studies also consistently report that people with lower perceived facial adiposity are rated as more attractive and healthier compared to people with higher perceived facial adiposity (Klaczynski et al., 2009; Rantala et al., 2013a; Han et al., 2016; Foo et al., 2017). A study by Foo et al. (2017) showed that facial adiposity was a better predictor of attractiveness compared to sexual dimorphism, averageness, and symmetry, for male faces. The researchers also found that, for females faces, facial adiposity squared and sexual dimorphism were the best predictors of female facial attractiveness, while facial adiposity was also the strongest predictor of perceived health for male faces, while sexual dimorphism was the strongest predictor of perceived health for female faces, with facial adiposity failing to reach statistical significance.

As mentioned previously, for a specific facial cue to serve as a valid cue to health, people need to reliably detect the cue and agree on its relationship to health or attractiveness. Some studies have reported that participants are able to reliably estimate a person's body fat percentage or body mass index (BMI) from facial cues alone (Fisher et al., 2013; Tinlin et al., 2013; Han et al., 2016; Phalane et al., 2017). The fact that participants are able to accurately detect changes in BMI likely stem from the fact that changes in BMI have been linked to a predictable set of morphological characteristics such as width-to-height ratio, perimeter-to-area ratio, cheek-to-jaw-width ratio (Coetzee et al., 2010), shape of lower face outline, nose width, and eyebrow position (Mayer et al., 2017). A study by Re et al. (2013) reported that people can detect changes in BMI as small as 1.3 kg/m^2^ in male faces and 1.6 kg/m^2^ in female faces from facial cues alone. Another study by Re and Rule (2016b) corroborated this finding by reporting that an average change in BMI of 1.33 kg/m^2^ was sufficient for participants to report a noticeable change in the appearance of faces. In recent years there has also been a concerted effort from researchers to develop computer vision methods (Wen and Guo, 2013; Kocabey et al., 2017; Barr et al., 2018) and statistical models (Wolffhechel et al., 2015; Stephen et al., 2017) to predict BMI from facial images.

Being able to draw inferences about a person's weight from facial cues of adiposity, provides a potentially robust perceptual link between facial cues and actual health. Body fat percentage and BMI has consistently been linked to various negative health outcomes. Overweight individuals are subject to unfavorable health outcomes including diabetes, mental health problems, impaired immune function, high and low blood pressure and heart disease (Kopelman, 2007; Dixon, 2010; Zaccardi et al., 2017); while being severely underweight could be an indication that a person is vulnerable to communicable diseases (Fisher et al., 2014b), since being underweight is linked to malnutrition and compromised immune function (Ritz and Gardner, 2006; Dobner and Kaser, 2018). Accurate estimation of overall body weight from facial cues can thus serve as an effective cue to health, as lower or higher than average body weight could be indicative of a person's past, current and future health problems (Coetzee et al., 2009; Henderson et al., 2016). In addition to the strong relationship between BMI and health outcomes, a study by Levine et al. (1998) has also established a strong link between cheek adipose tissue and visceral abdominal fat and a study by Lee and Kim (2014) established a correlation between the distances between the inferior ear lobes and visceral fat. Inferring visceral fat from facial cues is a potentially important predictor of health outcomes, as visceral fat has been connected to negative health outcomes due to its high metabolic activity (Després and Lemieux, 2006). BMI is also highly hereditable, as twin studies have estimated the hereditability of BMI to be as high as 0.57 in people between 20 and 29 years of age, even after environmental variation and cultural-geographic regions were accounted for (Silventoinen et al., 2017).

Tables 1, 2 present summaries of studies investigating the relationship between facial adiposity, attractiveness, and perceived health.

**Table 1:** Summary of studies that investigated the relationship between facial adiposity and attractiveness.

**Study (First Author)/Year**	**Stimuli**			**Results**
	***N***	**Gender**	**Mean Age**	
Coetzee et al., 2009	84	M + F	<30	Significant curvilinear relationship between facial adiposity and attractiveness (*R^2^* = 0.186)
Klaczynski et al., 2009	64	M + F	<18	Average weight facial drawings were considered more attractive than obese facial drawings ($\eta_{p}^{2\;}=$ 0.91)
Coetzee et al., 2011	96	M + F	<30	On average, female faces were transformed by female raters to represent a BMI of 19.76 kg/m^2^ to reflect optimal attractiveness On average, female faces were transformed by male raters to represent a BMI of 20.01 kg/m^2^ to reflect optimal attractiveness
Coetzee et al., 2012	45	F	<30	Significant negative relationship between adiposity and attractiveness (*r* = −0.216)
Rantala et al., 2013a	52	F	<20	Negative correlation between facial adiposity and attractiveness (*r* = −0.481) No significant relationship between Hepatitis B antibody response and attractiveness ratings
Fisher et al., 2013^*^	100	M + F	<30	Small negative correlation between facial adiposity and attractiveness for males (*r* = −0.14) and females (*r* = – 0.19)
Re and Perrett, 2014	20	M + F	<30	On average, female faces were transformed to represent a BMI of 18.19 kg/m^2^ to reflect optimal attractiveness On average, male faces were transformed to represent a BMI of 22.46 kg/m^2^ to reflect optimal attractiveness Female faces were also transformed to a significantly lower BMI to maximize attractiveness compared to male faces ($\eta_{p}^{2}$ = 0.78)
Batres and Perrett, 2014	10	M + F	<30	Participants without internet access preferred faces with higher adiposity for females ($\eta_{p}^{2}$ = 0.170), but not for males ($\eta_{p}^{2}$ = 0.0005)
Fisher et al., 2014b	160	M + F	-	Two way interaction revealed that facial adiposity had a larger impact on facial attractiveness ratings (*d* = 4.7) compared to health judgments (*d* = 3.76)
Fisher et al., 2014a*	100	M + F	<30	Small negative correlation between facial adiposity and attractiveness for males (*r* = −0.16) and females (*r* = −0.13)
Han et al., 2016	96	F	<30	Significant negative relationship between adiposity and attractiveness (*r* = −0.59)
Re and Rule, 2016b	40	M + F	<30	On average, female faces were transformed to represent a BMI of 19.11 kg/m^2^ to reflect optimal attractiveness On average, male faces were transformed to represent a BMI of 23.79 kg/m^2^ to reflect optimal attractiveness
Foo et al., 2017	101	M	<30	Significant negative relationship between facial adiposity and attractiveness (*r* = −0.50)
	80	F	<30	Significant negative relationship between facial adiposity and attractiveness (*r* = −0.58)
Batres et al., 2017	10	M + F	<30	Participants from rural areas in Malaysia preferred faces with higher adiposity for females (*d* = 0.69), but not for males (*d* = 0.15). Participants from rural areas in El Salvador preferred faces with higher adiposity for females (*d* = 0.97), but not for males (*d* = 0.13).
Phalane et al., 2017	92	M	<30	Significant curvilinear relationship between adiposity and perceived health (*R*^2^ = 0.138)
Windhager et al., 2018	–	F	–	Curvilinear relationship between facial adiposity and attractiveness for male raters: adolescents (*R*^2^ = 0.22), young adults (*R*^2^ = 0.44) and older adults (*R*^2^ = 0.29).
	–	F	–	Curvilinear relationship between facial adiposity and attractiveness for female raters: adolescents (*R*^2^ = 0.24), young adults (*R*^2^ = 0.46) and older adults (*R*^2^ = 0.33).

* Used the same facial stimuli panels

**Table 2:** Summary of studies that investigated the relationship between facial adiposity and perceived health.

**Study (First Author)/Year**	**Stimuli**			**Results**
	**N**	**Gender**	**Mean Age**	
Coetzee et al., 2009	84	M + F	<30	Significant curvilinear relationship between adiposity and perceived health (*R*^2^ = 0.263)
Coetzee et al., 2011	96	F	<30	Female raters indicated significantly lower BMI is required for attractiveness (19.76 kg/m^2^) compared to optimum health (20.84 kg/m^2^). No such difference was found for males
Fisher et al., 2014b	20	M + F	-	Facial adiposity had a significant effect on perceived health ratings (*d* = 3.76)
Han et al., 2016	96	F	<30	Significant linear relationship between adiposity and perceived health (*r* = −0.52)
Stephen et al., 2017	60	M + F	-	Raters significantly reduced BMI (*d* = 3.56) and percentage body fat (*d* = 3.96) to enhance healthy appearance
Axelsson et al., 2018	16	M + F	-	Higher sickness ratings were related to a more swollen facial appearance (*Z* = 4.1, *p* < 0.001)
Henderson et al., 2016	118	M + F	<30	Significant quadratic relationship between adiposity scores and perceived health ratings (*R*^2^ = 0.25)
	67	F	<30	Significant quadratic relationship between adiposity scores and perceived health ratings (*R*^2^ = −0.35)
Foo et al., 2017	101	M	<30	Significant negative relationship between facial adiposity and perceived health (*r* = −0.56)
	80	F	<30	Significant negative relationship between facial adiposity and perceived health (*r* = −0.40)
Phalane et al., 2017	92	M	<30	Significant curvilinear relationship between adiposity and perceived health (*R*^2^ = 0.138)
Windhager et al., 2018	5	F	–	Curvilinear relationship between facial adiposity and perceived health for male raters: adolescents (*R*^2^ = 0.13), young adults (*R*^2^ = 0.28) and older adults (*R*^2^ = 0.13)
	5	F	–	Curvilinear relationship between facial adiposity and perceived health for female raters: adolescents (*R*^2^ = 0.13), young adults (*R*^2^ = 0.30) and older adults (*R*^2^ = 0.18)

While research into the link between facial adiposity and health and attractiveness has been gaining momentum over the last decade, there are some challenges faced by researchers. Investigating the relationship between facial adiposity, actual health, and attractiveness provides a series of methodological challenges. As both underweight and overweight individuals are likely to be judged less healthy and attractive, Coetzee et al. (2009) proposed that the relationship between perceived health, attractiveness and adiposity is best represented by a quadratic relationship. Seeing as both overweight and significantly underweight individuals are subject to negative health outcomes, any large deviations from average levels of adiposity is likely to lead to decreases in health and attractiveness ratings. Subsequent studies confirmed that curvilinear models are often the best fitting models for these data (Phalane et al., 2017; Windhager et al., 2018), though this is not always the case (Coetzee et al., 2012; see for example Foo et al., 2017). In studies where sampling range restrictions occur for BMI, it is also possible that the range restriction obscures the quadratic pattern of the results. One notable study by Windhager et al. (2013) created a set of female adolescent geometric morphs that allowed the researchers to produce a set of 5 images which ranged from −5 *SD* (19% body fat) to +5 *SD* (64% body fat). These facial morphs were then used in a recent study where 274 male and female participants had to rate each of the 5 images on maturity, dominance, masculinity, perceived health and attractiveness (Windhager et al., 2018). Analysis revealed that both health and attractiveness consistently produced distinct curvilinear shapes across all rater categories and genders.

In addition to the complex statistical relationship that exists between facial adiposity and preference ratings, there is also evidence to suggest that humans integrate information from multiple facial cues to evaluate faces. One of the facial cues that have been identified as playing a particularly important role in mediating the relationship between facial adiposity and health ratings is skin condition. According to Fisher et al. (2014b) judgments of health and attractiveness from facial adiposity in isolation produces a conundrums for raters, since it becomes very difficult to differentiate between individuals with low levels of facial adiposity due to health and those that have low facial adiposity levels due to ill-health. One way around this problem is to integrate information from skin condition with facial adiposity to provide more information on the potential risk associated with the facial adiposity levels being perceived. An experiment conducted by Fisher et al. (2014b) provided evidence that participants likely integrate both skin color and facial adiposity cues when judging faces for health and attractiveness. Redness and yellowness in the face has been associated with perceptions of health (Stephen et al., 2009, 2011) and in the study by Fisher et al. (2014b) there was evidence to suggest that participants relied more on color cues to decide if a face looked healthy or attractive when facial adiposity levels were relatively low.

According to the World Health Organization (WHO) classification, a healthy BMI for an average adult is between 18.5 and 24.9 kg/m^2^ (World Health Organization, 2018). Numerous studies have shown that people generally prefer facial adiposity levels that are associated with the normal range of the World Health Organization's classification for BMI and that lower levels of facial adiposity is typically rated as more attractive, even across cultures (Coetzee et al., 2011, 2012, 2014; Re et al., 2011; Re and Rule, 2016b). For example, a study by Coetzee et al. (2011) found that, female participants judging other female faces, indicated that facial adiposity levels associated with a BMI of 19.76 kg/m^2^ are optimally attractive, while male raters indicated that adiposity levels associated with a BMI of 20.01 kg/m^2^ are optimally attractive for female faces. A similar study by Re and Rule (2016b) found that participants preferred facial adiposity levels associated with a BMI of 19.11 kg/m^2^ for female faces and 23.79 kg/m^2^ for male faces. Coetzee (2011) compared facial adiposity preferences between a cohort of British and African participants and found that Caucasian males, African females and African males all preferred similar levels of facial adiposity for attractiveness and health. Despite the evidence that there is agreement across cultures on facial adiposity levels associated with health and attractiveness, there does appear to be some degree of divergence in BMI preference judged from facial adiposity and BMI preferences as judged from bodies across cultures. For example, a study by Tovée et al. (2006) found that ethnic South African Zulus preferred a BMI that is above the upper limit of the normal range for both health and attractiveness when judging female bodies. Since most studies on facial adiposity preferences utilize university students, Coetzee et al. (2012) argues that this discrepancy could potentially be attributed to repeated exposure to Western media ideals and improved access to resources that African university students enjoy. This line of reasoning is also supported by Tovée et al. (2006) who found that South African born Zulus who immigrated to the United Kingdom preferred a lower BMI for both attractiveness and health compared to Zulus still residing in South Africa (also see Tovée et al., 2007). At present, more research is needed to elucidate the cultural and environmental factors associated with facial adiposity preferences across cultures.

There also appears to be relative differences between males and females when it comes to judgments of facial adiposity levels required for a healthy appearance for female faces. Coetzee et al. (2011) report that females preferred a slightly lower BMI (19.76 kg/m^2^) for attractiveness compared to optimum health (20.84 kg/m^2^) when rating other females, while no such difference was found for males. One hypothesis that has been put forth to account for this discrepancy, is that socio-cultural factors related to judgments of body fat influences and shapes female body weight ideals, especially via media exposure to a thin ideal (Groesz et al., 2002; Harrison and Hefner, 2006; Coetzee et al., 2011). Though the link between facial adiposity and perceived health and attractiveness appears to be remarkably stable, facial adiposity likely interacts in complex ways with various biological, environmental and socio-cultural factors, as well as other facial cues such symmetry, skin color, skin texture, sexual dimorphism and averageness in shaping social and health perceptions derived from faces.

### Facial Adiposity and Health Outcomes

One of the primary reasons why researchers are interested in facial adiposity is its potential to act as a robust cue to health outcomes. Given the strong relationship that exists between facial adiposity and BMI, negative health outcomes related to BMI should therefore also be linked to facial adiposity. To date, numerous studies have found a relationship between facial adiposity and actual health outcomes, although the results are not always consistent.

#### Cardiovascular Health

A study by Coetzee et al. (2009) found that facial adiposity was associated with systolic blood pressure (*r* = 0.28) and diastolic blood pressure (*r* = 0.43), as well as a cardiovascular-illness component (*r* = 0.41). In addition to the results reported by Coetzee et al. (2009) a study by Reither et al. (2009) also found a link between perceived facial adiposity and cardiovascular health. Reither et al. (2009) used 3,027 yearbook photographs of teenagers from the Wisconsin Longitudinal Study (WLS), which also captured basic health data from 1957 to 2004. The researchers found that increased facial adiposity ratings in adolescent faces were associated with high blood pressure (*OR* = 1.25), heart trouble (*OR* = 1.20) and heart disease (*OR* = 1.69) later in life. Recently a study by Stephen et al. (2017) also reported that a geometric morphometric model that captured aspects of facial shape from images, could be used to predict 21% of the variance in blood pressure in their sample. These results indicate that there is a robust link between cardiovascular health and facial adiposity.

#### Immunity

Coetzee et al. (2009) reported that higher levels of rated facial adiposity was associated with an increase in the number of colds and flu's participants reported (*r* = 0.209), bout length of those reported colds and flu's (*r* = 0.24), the frequency of antibiotics use reported by participants (χ^2^ = 11.706) and a respiratory-illness component (*r* = 0.29). These relationships remained significant, even after the researchers controlled for parental income and age. A study on male participants by Rantala et al. (2013a) found an association between perceived facial adiposity and immunity (β = −0.38) using a direct measure of immune function (antibody response to a Hepatitis B vaccination). The researchers also reported that perceived facial adiposity mediated the relationship between antibody response and facial attractiveness. However, a similar study using female participants, found no link between the antibody response produced by a Hepatitis B vaccination and percentage body fat, though it should be mentioned that no direct measures of facial adiposity were included in the study (Rantala et al., 2013b). Although the studies reported above found indications that immune function may be linked to facial adiposity, especially in males, other studies have not produced the same pattern of results when using different measures of immune function. For example, a study by Phalane et al. (2017) using African participants found no significant relationship between adiposity and immune responsiveness as measured by functional cytokine profile and C-reactive proteins in African men. It is worth mentioning, however, that the sample size for the cytokine profile was relatively low (n = 41) and could thus reflect a potential type II error due to a lack of statistical power. A recent study by Foo et al. (2017) also found no relationship between facial adiposity and immune function (bacterial killing capacity, overall bacterial immunity, bacterial suppression capacity, and lysozyme activity) in either males or females. To date, there is some evidence to suggest that facial adiposity and immune function are linked, especially in men, although more research is needed to clarify the potential relationship between different aspects of immune function and facial adiposity.

#### Mental Health

Relatively few studies have investigated the link between facial adiposity and mental health. A study by Martinson and Vasunilashorn (2016) that was conducted using the same basic methodology applied to the WLS data used by Reither et al. (2009), found that females who were rated as being overweight (*M* = 8.79) in 1957 scored significantly higher on the Center for Epidemiological Studies Depression Scale (CES-D) later in life compared to normal weight females (*M* = 6.94). No statistically significant difference was found between overweight (*M* = 6.40) and normal weight (*M* = 5.97) CES-D scores for male students. Additionally, a study conducted by Tinlin et al. (2013) using female participants, also found that rated facial adiposity was negatively correlated with a psychological condition factor (*r* = 0.29). These findings suggest that facial adiposity may also serve as a valid cue to mental health in females, although more research is required to draw any conclusions about the link between mental health and facial adiposity in males.

#### Hormones

The relationship between facial adiposity and various hormone levels has also produced largely mixed results. For example, a study by Tinlin et al. (2013) conducted using female participants reported a significant negative correlation between salivary progesterone levels (*r*_s_ = −0.30) and facial adiposity, but found no relationship between facial adiposity and estradiol levels, though the results should be interpreted with caution due to the small sample size (*n* = 49). Rantala et al. (2013a) found a significant relationship between circulating testosterone and facial adiposity in men (*r* = −0.52). Han et al. (2016) found no relationship between facial adiposity and cortisol levels in females, while Rantala et al., 2013b also found no relationship between cortisol levels and percentage body fat in females. To date, most of the research done on the relationship between hormones and facial adiposity has been done using female participants.

#### Other Health Outcomes

In addition to cardiovascular health, immune function, mental health and hormone levels, some studies also investigated potential links between facial adiposity and other health outcomes such as diabetes and arthritis or indicators of health such as oxidative stress and sperm health. For example, Reither et al. (2009) found that adolescents who were rated as having higher levels of facial adiposity were also more likely to experience muscle aches (*OR* = 1.13), shortness of breath (*OR* = 1.10) and chest pains (*OR* = 1.21) during the course of their lives, but also showed an increased risk of developing arthritis (*OR* = 1.19), diabetes (*OR* = 1.44), and die of non-accidental causes (*OR* = 1.32). A study by Foo et al. (2017) investigated the relationship between facial adiposity and oxidative DNA damage, as well as level of lipid peroxidation. The researchers found a significant relationship (*r* = −0.22) between facial adiposity and urinary *8-hydroxy-2*′ –*deoxyguanosine* (8-OHdG), a biomarker for oxidative stress and carcinogenesis (Valavanidis et al., 2009) for males, but not for females. However, Foo et al. (2017) also reported that no relationship existed between facial adiposity and a measure of lipid peroxidation (isoprostane levels) in either males or females. Lastly, Foo et al. (2017) found no relationship between facial adiposity and sperm health (rapid progressive motility, linearity of sperm movement, sperm concentration and percentage motile sperm) for males. A study by Tinlin et al. (2013) investigated the relationship between facial adiposity and general health markers in female participants. The researchers found that rated facial adiposity was not correlated with a physical condition factor, although the researcher did note that there was evidence for high collinearity between the physical condition and psychological condition factors in their study. A combination of these factors (labeled general condition factor), displayed a moderate association with facial adiposity (*r* = 0.41).

When combined, the results from these studies indicate that facial adiposity can serve as a reliable indicator of actual health outcomes including immune function, cardiovascular health, respiratory health, mental health and oxidative stress, although more research is needed to elucidate the link between these health outcomes and facial adiposity. The next section briefly touches upon factors that can potentially influence or mediate the relationship between facial adiposity and judgments of attractiveness and health.

Table 3 presents a summary of studies investigating the relationship between facial adiposity and health outcomes.

**Table 3:** Summary of studies that investigated the relationship between facial adiposity and health outcomes.

**Study (First Author)/Year**	**Stimuli**			**Result**
	**N**	**Gender**	**Mean Age**	
Coetzee et al., 2009	84	M + F	<30	Significant relationship between facial adiposity and cold and flu Number (*r* = 0.21), bout length (*r* = 0.24), frequent antibiotics use (χ^2^ = 11.71), respiratory-illness component (*r* = 0.29), systolic blood pressure (*r* = 0.28), diastolic blood pressure (*r* = 0.43), cardiovascular-illness component (*r* = 0.41)
Reither et al., 2009	3027	M + F	<30	Adolescents who were rated as having higher levels of facial adiposity were more likely to experience muscle aches (*OR* = 1.13 [95% CI = 1.02, 1.26]), shortness of breath (*OR* = 1.10 [95% CI = 0.93, 1.31]) and chest pains (*OR* = 1.21 [95% CI = 0.96, 1.53]). Adolescents who were rated as having higher levels of facial adiposity were also more likely to develop arthritis (*OR* = 1.19 [95% CI = 1.08, 1.31]), high blood pressure (*OR* = 1.25 [95% CI = 1.11, 1.41]), diabetes (*OR* = 1.44 [95% CI = 1.09, 1.90]) and heart trouble (*OR* = 1.20 [95% CI = 0.96, 1.51]) and die of non-accidental causes (*OR* = 1.32 [95% CI = 1.10, 2.58]) or heart disease (*OR* = 1.69 [95% CI = 1.10, 2.58]).
Tinlin et al., 2013	50	F	<30	Significant correlation between facial adiposity and a psychological condition factor (*r* = 0.29), as well as salivary progesterone (*r_*s*_* = −0.30). No relationship between facial adiposity and a physical condition factor (*r* = 0.26) or estradiol levels (*r_*s*_* = −0.18)
Rantala et al., 2013a	69	M	<30	Circulating testosterone was positively correlated with adiposity (*r* = 0.52) Hepatitis B anti-body response was also significantly associated with facial adiposity (β = −0.38)
Han et al., 2016	96	F	<30	No Significant relationship between adiposity and average cortisol levels (*r* = 0.05)
Martinson and Vasunilashorn, 2016	4410	M + F	<30	Females who were rated as being overweight (*M* = 8.79) scored significantly higher on the CES-D depression inventory compared to females who were rated as normal weight (*M* = 6.94) There was no statistically significant difference between CES-D scores for overweight (*M* = 6.40) and normal weight (*M* = 5.97) males
Foo et al., 2017	101	M	<30	Significant negative correlation between facial adiposity and an indicator of oxidative stress (8OHdG levels) (*r* = −0.22). No relationship between facial adiposity and an indicator of lipid peroxidation, immune function (bacterial killing capacity, overall bacterial immunity, bacterial suppression capacity and lysozyme activity), or semen quality (rapid progressive motility, linearity of sperm movement, sperm concentration and percentage motile sperm)
	80	F	<30	No relationship between facial adiposity and indicators of oxidative stress and lipid peroxidation or immune function (bacterial killing capacity, overall bacterial immunity, bacterial suppression capacity and lysozyme activity)
Phalane et al., 2017	92	M	<30	No significant relationship between adiposity and immune function as measured by a cytokine component (*r* = 0.282) or C-reactive protein (*r* = −0.0007).

### Factors That Potentially Contribute to Adiposity Preferences

While numerous studies have found a robust link between facial adiposity and perceptions of health and attractiveness, a number of potential mediating factors have been identified that could serve to modify our facial adiposity preferences. A study by Re et al. (2011) demonstrated that our preferences for particular adiposity levels can be altered, at least in the short term. First, the researchers used a pre-test to establish a baseline for participants' facial adiposity preferences. By exposing participants to either “plus-sized” female bodies or regular female bodies, the researchers could induce an after-effect where participants who viewed the “plus-size” bodies increased their adiposity preference by an average of 0.5 kg/m^2^. Participants thus preferred faces higher in adiposity after viewing the “plus-size” bodies. This finding illustrates an adaptation effect, whereby exposure to certain stimuli can create a preference shift toward that particular type of stimulus. According to the authors, this after-effect could be an indication that overall attractiveness judgments require integration of multiple cues, though these cues are often investigated independently.

The idea of an adaptation effect is also related to repeated exposure to facial cues found in our own ethnicity, or ethnicities that we are regularly exposed to. In a study by Schneider et al. (2013) Japanese and German participants were asked to provide body weight estimates for both German and Japanese individuals based on facial photographs alone. The researchers found that German observers tended to slightly overestimate the body weight of the Japanese faces, while the Japanese participants significantly underestimated the body weight of the German faces. The researchers argue that one potential explanation for the gross underestimation of body weight for German faces by Japanese participants, is that Japanese participants utilized an inappropriate reference point regarding the link between facial shape and body weight for German faces. Since Japanese faces tend to display more rounded and broad (brachycephalic) head proportions, German faces associated with an average body weight appeared more slender, compared to a Japanese face associated with and average body weight, which Japanese participants would have been more familiar with. These findings imply that accurate weight perceptions derived from facial cues, is also contingent upon reasonable exposure and familiarity with the association between facial phenotypic variation and body weight of a particular ethnic group.

Another factor that can play a significant role in influencing people's adiposity preferences, is potential pathogen exposure and how it relates to our behavioral immune system. Studies have shown that levels of pathogen disgust sensitivity can have a dramatic influence on individuals' perceptions of facial cues associated with attractiveness or perceived health (Park et al., 2012; Jones et al., 2013). These result suggest that a pathogen disgust reaction may indeed play a role in our relative tolerance for facial cues that may signal ill-health. A study by Fisher et al. (2013) found that men with higher pathogen disgust responses showed preferred facial cues associated with lower weight, indicating that individual differences in pathogen disgust also play a role in relative levels of facial adiposity that people find attractive or perceive to be healthy. These result are also supported by Rantala et al. (2013a) who found that facial adiposity mediated the association between facial attractiveness and immune response in men. However, a study by Dixson et al. (2017) found no relationship between facial adiposity preferences and malarial prevalence for people living in urban or rural areas in Vanuatu, a series of small Pacific islands. These results suggest that the relationship between facial adiposity preferences and pathogen exposure or disgust, while theoretically elegant, have to be interpreted with caution.

Environmental pressures also appear to play a role in shaping our preferences for facial or body adiposity. For example, it has been demonstrated that resource scarcity could play a role in males' preference of breast size, with men from lower socio-economic regions preferring larger breasts, due to breast size being a good indicator of adipose tissue reserves (Dixson et al., 2011; Swami and Tovée, 2013). A study by Batres and Perrett (2014) found that people in rural areas of El Salvador tend to prefer female faces with higher levels of adiposity, compared to people from urban areas. One hypothesis that has been proposed to account for this difference is the fact that rural areas, especially in developing nations, tend to be poorer and people from these areas tend to have less access to resources such as food or medicine. A similar study conducted by Swami and Tovée (2007) also provide evidence that people from resource-poor areas of Thailand preferred female bodies that were higher in BMI compared to people from more industrialized areas of Thailand. A more recent study by Batres et al. (2017) replicated the results of Batres and Perrett (2014) in another cohort of people from El Salvador, as well as Malaysia. What is especially interesting about these results is the fact that a difference in preference for facial adiposity levels between more developed and poorer areas was only found for females faces. One possible reason for this is that a trade-off exists in poorer areas, where males have to weigh the potential negative long-term health outcomes associated with higher levels of adiposity and more immediate concerns regarding survival and reproductive fitness of females (Batres et al., 2017). Another possible contributor to this phenomenon could be increased media exposure to thin ideals in more developed areas, which then serves to alter people's baseline expectations of attractiveness or health, via and adaptation effect as described by Re et al. (2011) and Batres and Perrett (2014). It should be pointed out, however, that there are studies that problematize the view that people in resource-poor areas prefer bodies or faces associated with a slightly higher BMI. For example, a study by Dixson et al. (2017) found no evidence to support the hypothesis that males from rural area would prefer females faces associated with higher levels of facial adiposity. A series of studies conducted in China, Papua New Guinea, Cameroon, Indonesia, Samoa and New Zealand, also found a high degree of cross-cultural consensus when participants were asked to judge female body attractiveness, with both males and females preferring a lower waist-to-hip ratio (WHR) regardless of BMI (Dixson et al., 2010a,b; Singh et al., 2010). These results suggest that sexually dimorphic fat distribution could in fact be more important than overall BMI when judging female body attractiveness, even across ethnicities or socio-economic regions.

A study by Weston et al. (2015) revealed that judgments of weight in faces can even be influenced by the expression of the facial stimuli. The researchers observed that participants tended to rate male faces containing sad expressions as more overweight compared male faces containing a neutral facial expression. A study by Henderson et al. (2016) also found that downward mouth curvature was negatively correlated with perceived health (*r* = −0.20) and upward mouth curvature was positively correlated with perceived health (*r* = 0.51), presumably because a frown is associated with a sad facial expression that reflect a negative mood or mental state. It is thus likely that facial cues are also evaluated within the context of emotional attributions, adding yet another layer of complexity to the way in which we judge physical and emotional health from facial cues in others.

### Methodological Considerations in the Study of Facial Adiposity

In the laboratory there are several factors related to the facial stimuli that can influence the outcome of a study. For example, a study by Jones et al. (2012) found that perceptions and the derived judgments of facial stimuli can be altered by changing the viewing angle of the face. According to the authors, this effect is likely attributable to directional asymmetries in the perception of facial cues related to shape. A study by Russell et al. (2016) found that facial contrast can also influence perceptions of health within the face, with faces low in contrast being judged as less healthier. These two studies highlight the importance of controlling for subtle factors such as viewing angle and facial contrast in the generation of facial stimuli, as they could potentially confound the results of the study.

In addition to the way in which stimuli are generated, statistical and research design considerations also need to be taken into account when conducting facial morphology research. For example, Windhager et al. (2018) proposes that a major drawback of the traditional reliance on *p*-values in the analysis of data derived from facial stimuli, is the fact that facial morphology research often involves complex nested designs. Most designs in the field use human raters to rate multiple facial stimuli on one or more dimensions. Researchers then often calculate a mean rating for a particular face and although inter-rater reliability is mostly high (α > 0.80), the variance associated with rater responses is usually lost when rater responses are collapsed to a single mean. Many studies also use suboptimal sample sizes (*n* > 100) due to the complexity of the designs, which can lead to a lack of statistical power in detecting smaller effect sizes (see for example Tinlin et al., 2013; Phalane et al., 2017). In reality, collinearity concerns are likely always going to be a potential problem researchers will have to deal with, due to the complex facial cue integration that happens within an organic judgment faces. However, recent advances in the creation of facial morphs can potentially allow researchers to manipulate facial cue dimensions with increasing precision, while also theoretically isolating specific facial cues within a panel of facial stimuli (Windhager et al., 2013, 2018; Re and Perrett, 2014). Bottom-up or data-driven methodologies can also offer researchers a way to increase the precision with which facial cues are specified by reducing shape coordinates to a set of principle components that can be used in further analysis (Wolffhechel et al., 2015; Henderson et al., 2016).

### Estimating BMI From Facial Adiposity

One of the key components of utilizing weight related facial cues to make inferences about people's health is that people should be able to accurately judge body mass from facial cues alone. To our knowledge no study has provided a quantitative synthesis of the link between facial adiposity and judgments of body weight across different studies published in the field. A meta-analysis was therefore conducted to evaluate the strength of the relationship between judgments of facial adiposity and BMI/ percentage body fat.

## Methods

### Inclusion Criteria

All peer-reviewed journal articles that provided an effect size that quantifies the relationship between perceived facial adiposity and estimations of BMI or percentage body fat were included in the meta-analysis. Studies that reported more than one effect size based upon the same facial stimuli were excluded from the analysis due to potential biases in heterogeneity estimates. For example, a study by Coetzee et al. (2012) calculated a correlation coefficient for the relationship between perceived facial adiposity and BMI, as well as percentage body fat for the same participants. Although two effect sizes can be extracted from this study, the two effect sizes obtained from the same group would not be statistically independent. Similarly, Fisher et al. (2014a) used the same facial stimuli as Fisher et al. (2013), thus the effect sizes obtained from these two studies would also not be statistically independent. The effect size for the relationship between facial adiposity and percentage body fat from Coetzee et al. (2012) and the study by Fisher et al. (2014a) were thus excluded from the analysis. No restriction criteria were placed on the age, gender or ethnicity of either the facial stimuli or raters in studies to be included in the meta-analysis.

### Search Strategy

**Figure 1:**
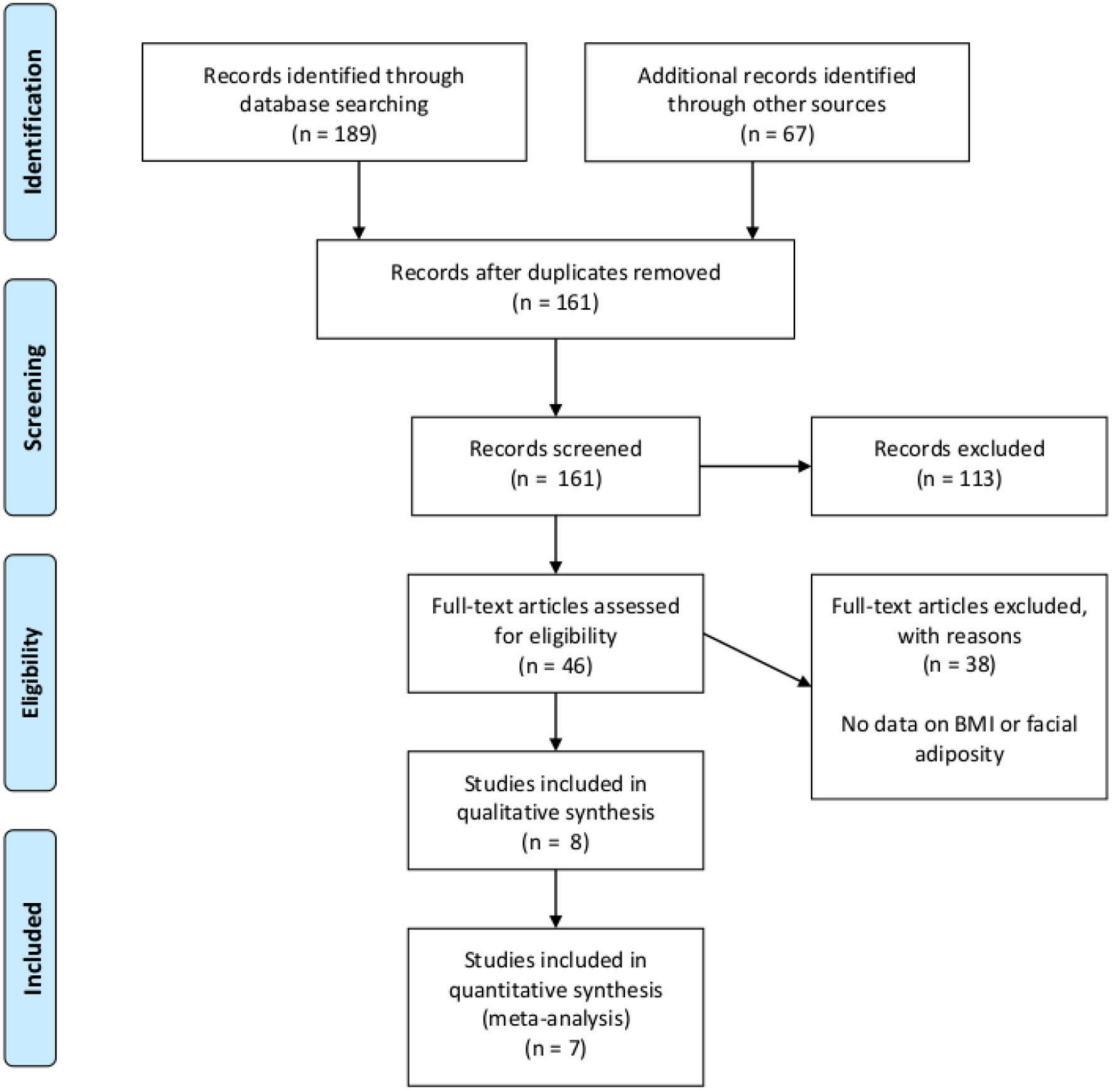
PRISMA flowchart for selection of studies.

A systematic review protocol was developed in accordance with the Preferred Reporting for Items for Systematic Reviews and MetaAnalyses-Protocols (PRISMA) (Moher et al., 2009). The PRISMA protocols can easily be reproduced by other researchers thereby ensuring transparency and reliability of meta-analyses (Gurevitch et al., 2018). The use of PRISMA protocols is becoming increasingly popular within the field of evolutionary psychology (Geniole et al., 2015; see for example Gouda-Vossos et al., 2018). In line with the protocol developed for this study, all studies that reported an effect size for the relationship between perceived facial adiposity and BMI or percentage body fat were identified by searching relevant databases for peer-reviewed articles that contained the phrase “facial adiposity”: Pubmed (9), EbscoHost (27), Science Direct (66), ProQuest (66), and Web of Science (21). Once databases were searched, reference lists of prominent published articles were also searched and any additional relevant abstracts were added. Results were entered into the open-source software management package Zotero Version 5.0. Duplicate entries were removed, after which abstracts were screened for relevance in line with the inclusion criteria by the authors. After the initial screening procedure, the full-text articles were downloaded for the remaining list and re-screened for relevance. See Figure 1 for the PRISMA flow diagram.

### Data Extraction

A data extraction template was used to capture all the relevant data from the articles selected for inclusion in the meta-analysis. The data extraction sheets included detailed information on the types of stimuli including gender, age, BMI/percentage body fat, ethnicity, as well as the rating scale used and if the facial stimuli were captured in 2D or 3D. Demographic information on the raters was included as well, including gender, age and ethnicity. Finally, effect sizes were captured for all relevant studies.

### Data Synthesis

All extracted effect sizes were converted to Pearson's *r* before any analyses were conducted. As discussed previously, studies often use the same facial stimuli or raters multiple times during a study, thus creating a complex nested design structure. This nested structure means that it is not unusual for a particular study to produce two or more analysis outcomes that were derived from highly correlated or identical sources within the study. If this statistical dependency is not adequately accounted for in the analysis procedures used by the researchers, they risk introducing bias in the variance estimation during the analysis (Hunter and Schmidt, 2004). In order to account for the within-study statistical dependence, we aggregated all effect sizes within each study to produce a single estimated effect size for that study using the “*Agg*” function contained in the “*Mac*” package for *R* (Del Re and Hoyt, 2012; R Core Team, 2013). As information on the true correlations of within-study effect sizes were unavailable, all within-study effect size correlations were fixed at 0.50 (Wampold et al., 1997). This aggregation procedure produced seven effect size estimates, one corresponding to each study. Due to key differences between studies regarding selection and presentation of facial stimuli, as well as sample composition, meta-analyses were conducted using random-effect models (Borenstein et al., 2010; Quintana, 2015). The aggregated Pearson's *r* for each study was transformed to a Fisher's *z* scale, as *r* is not normally distributed. These effect size estimates were back transformed where appropriate in the presentation of the results. See Table 4 for a summary of studies included in the meta-analysis.

**Table 4:** Summary of studies that investigated the relationship between facial adiposity and BMI/Percentage body fat.

**Study (First Author)/Year**	**Stimuli**				**Raters**			**Effect Size (*r*)**
	**n**	**Gender**	**Age**	**BMI**	**n**	**Gender**	**Age**	
Coetzee et al., 2009	84	M + F	*M =* 21.13	*M =* 22.95	55	M + F	-	0.66
Coetzee et al., 2012	45	F	*M =* 19.84	–	30	M + F	-	0.75
Tinlin et al., 2013	50	F	*M =* 20.3	*M =* 23.2	21	M + F	*M =* 21.1	0.68
	50	F	*M =* 24.3	*M =* 20.1	26	M + F	*M =* 23.2	0.66
	50	F	*M =* 19.74	*M =* 23.8	160	M + F	*M =* 23.77	0.63
Rantala et al., 2013b	69	M	*M =* 23	*M =* 17.36	14	F	*M =* 23.6	0.75
Fisher et al., 2013	50	M	*M =* 24.2	*M =* 20.1	50	M + F	*M =* 22.54	0.58
	50	F	*M =* 24.3	*M =* 20.1	50	M + F	*M =* 24.11	0.66
Han et al., 2016	96	F	*M =* 21.42	*M =* 25.53	463	M + F	*M =* 24.14	0.67
Phalane et al., 2017	92	M	*M =* 20.4	*M =* 21.7	20	F	*M =* 22.5	0.68

## Results

A random effects model weighted by sample size was performed using the “*metaphor*” package in R (Viechtbauer, 2010). The analysis revealed a strong positive overall correlation between perceived facial adiposity ratings and BMI/percentage body fat [*r* = 0.71; 95% CI (0.66, 0.76), *p* < 0.001]. Heterogeneity statistics revealed no statistically significant between-study heterogeneity [*Q* = 7.12 (df = 6), *p* = 0.31; *I*^2^ = 19.36%; τ^2^ 0.004; τ = 0.06]. According to guidelines published by Higgins et al. (2003), an *I*^2^ proportion below 25% is a good indicator of low between-studies variability and can serve as a more robust indicator of between-study variance in small sample sizes.

**Figure 2:**
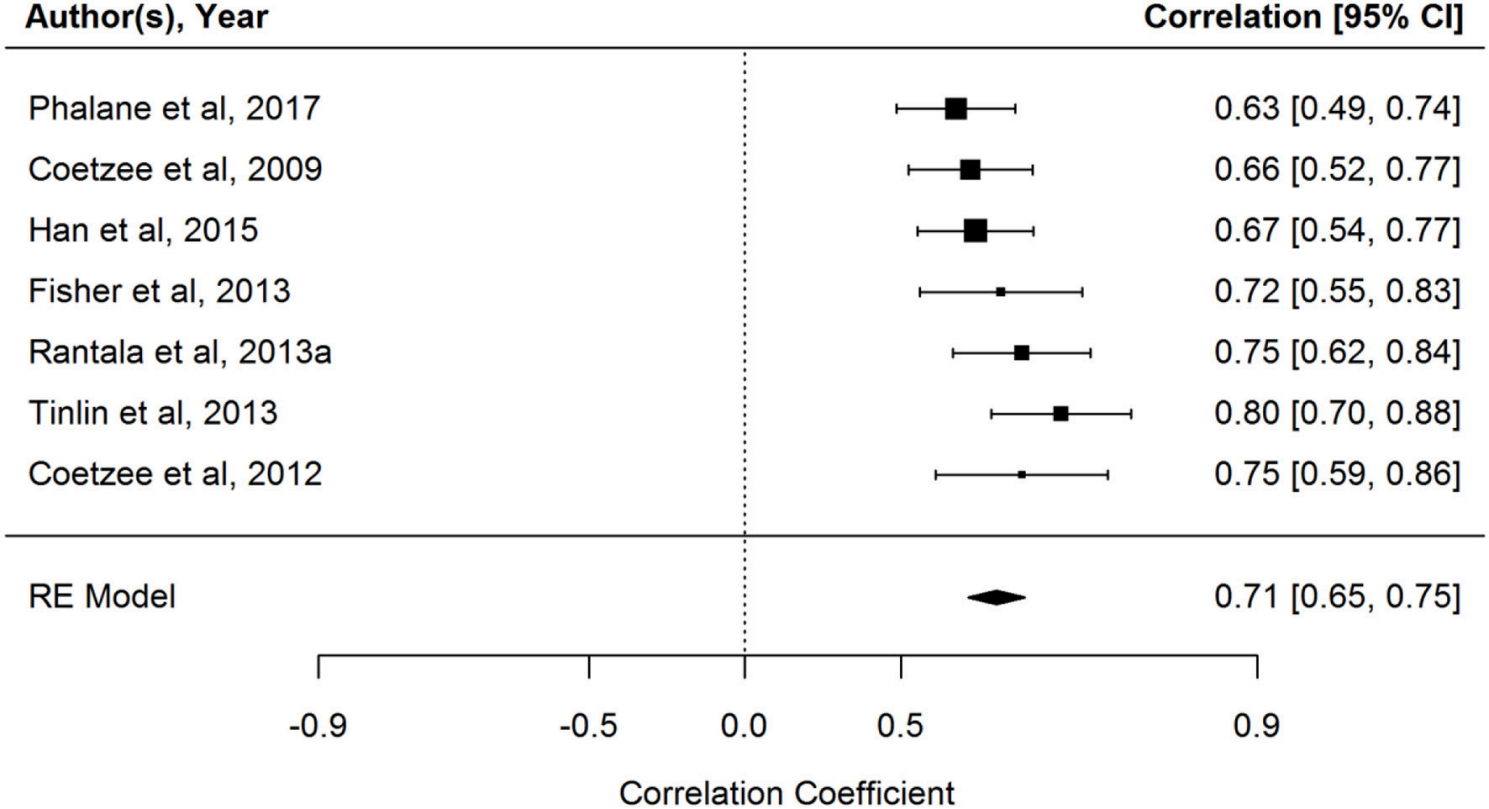
Forest plot of relationship between perceived facial adiposity and BMI/ percentage body fat.

**Figure 3:**
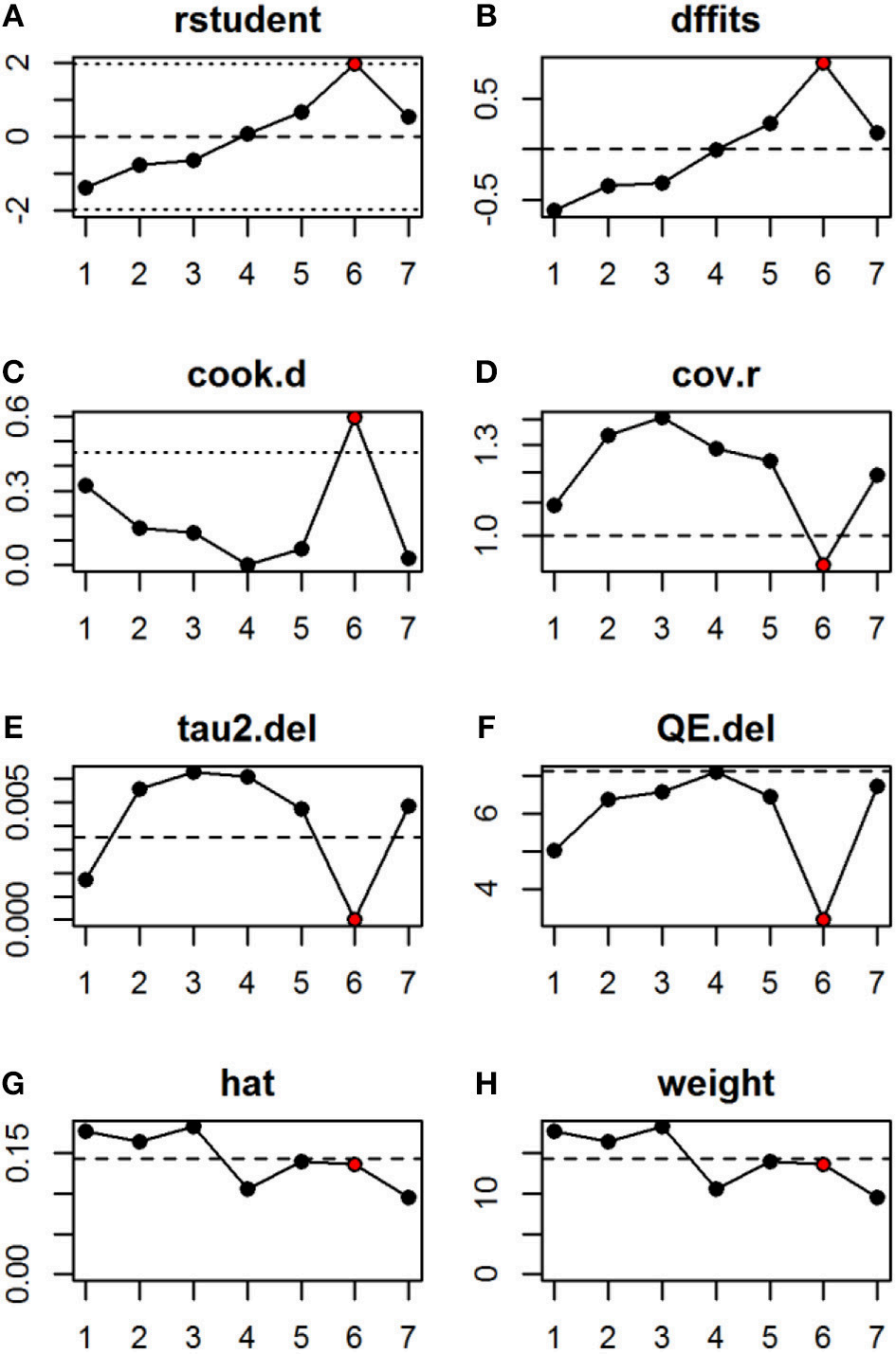
Plot of the **(A)** studentized deleted residuals; **(B)** DFFITS values; **(C)** Cook's distances; **(D)** covariance ratios; **(E)** estimates of τ2; **(F)** test statistics for (residual) heterogeneity; **(G)** hat values; and **(H)** weight for the 7 studies included in the analysis.

Sensitivity analysis was conducted to investigate if any of the studies included in the analysis contributed disproportionately to heterogeneity. Outlier and influential case diagnostics were conducted, which indicate that only Coetzee et al. (2012) (Study 6 in Figure 2) displayed signs of being a potential outlier or influential case (Viechtbauer and Cheung, 2010). See Figure 3 for influential case diagnostics. To test if the removal of Coetzee et al. (2012) from the analysis would influence the overall model fit, the study was excluded and the model re-fitted. The re-fitted model did not make a significant impact on the interpretation of the overall results however [*r* = 0.70; 95% CI (0.65, 0.76), *p* < 0.001]. Moderator analysis also revealed that the ethnicity of the facial stimuli (African or Caucasian) did not have a moderating effect on the relationship between perceived facial adiposity ratings and BMI/ percentage body fat [*Q* (1), 0.50, *p* = 0.48].

### Risk of Bias

**Figure 4:**
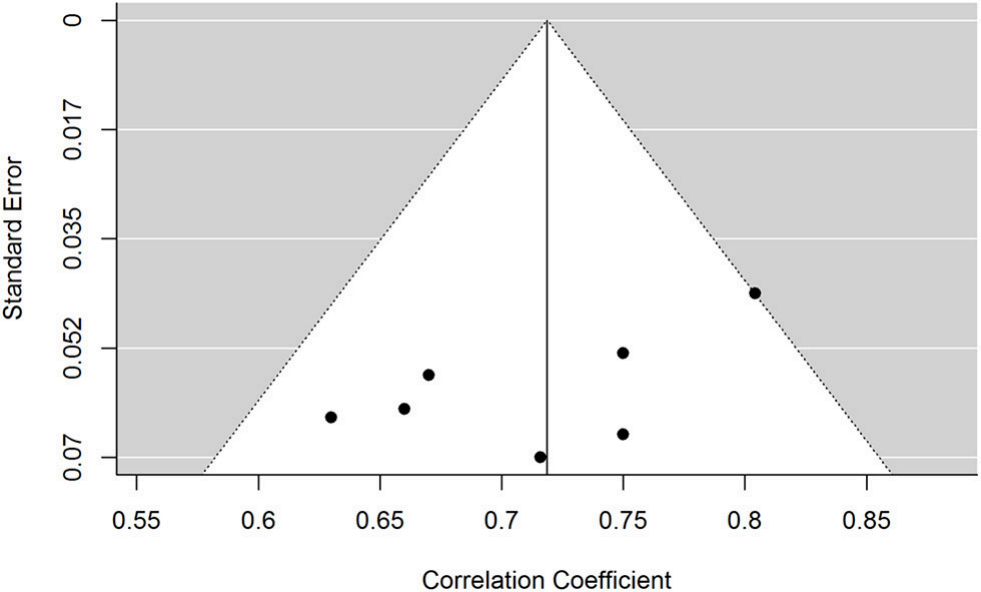
Funnel plot of standard errors by correlation coefficient.

Figure 4 shows a funnel plot of correlation coefficients plotted against standard errors for each study. Egger's regression test showed no evidence of any asymmetry in the funnel plot. Due to the relatively low sample size (*n* = 7) of the study, visual symmetry was hard to estimate and therefore no trim-and-fill methods were used.

## Discussion

Facial adiposity has consistently been linked to perceptions of attractiveness and health, with heavier faces being judged to be more unattractive and unhealthier. To date, facial adiposity has also been linked to a number of actual health outcomes including: cold and flu number, duration of colds and flu, frequency of antibiotic use, respiratory illness, blood pressure, cardiovascular illness, salivary progesterone, psychological well-being, arthritis, diabetes, circulating testosterone, immune function, and oxidative stress. While a strong relationship between facial adiposity, attractiveness, perceived health and actual health outcomes has been reported, there are a few limitations to the current evidence presented in favor of facial adiposity as an important contributor to health and attractiveness judgments. It is noteworthy that the majority of the studies published on facial adiposity used Caucasian students in their early to mid-twenties as both stimuli and raters, with isolated studies being done on Asian, Hispanic and African faces. A study by Batres and Perrett (2014) also found that people without internet access preferred female faces with higher levels of adiposity compared to people with internet access. This finding highlights a key shortcoming in the field, as most current published studies may provide a biased or incomplete picture of the way in which attractiveness and health judgments are made from facial cues related to body weight. It is therefore important for researchers to expand the diversity of sampled populations in future studies, as environmental pressures, and media exposure to Western weight ideals may differ substantially from region to region or culture to culture. It is also recommended that studies take socio-economic status of participants into account if possible.

There also appears to be important differences in judgments made by males and females regarding adiposity as a cue to health and attractiveness. For example, a link has been found between adiposity and immune responsiveness in male Rantala et al., 2013a, but not female faces Rantala et al., 2013b. There is also at least some evidence to suggest that Western body ideals may have a differential effect on males and females, where ideal attractiveness is associated with a lower BMI compared to ideal weight for females (Coetzee et al., 2011). Due to the fact that research in the field is highly reliant on cross-gender judgments of health and attractiveness, most studies would be well-advised to be sensitive to the nuanced differences in how people of opposite genders judge one another based on facial cues related to adiposity, especially across socio-economic or cultural boundaries.

In addition to the socio-economic, cultural and gender factors, researchers also need to pay close attention to stimuli generation and study designs when conducting research. The recent development of 3D facial imaging technology allows researchers to produce 3D facial images that more closely approximate the way people would be able to view another person's face in reality. This is especially important when participants are required to make judgments about facial shape, as 2D facial images contain less overall facial shape information compared to 3D images. Researcher also need to be mindful of the potential drawbacks of using traditional statistical procedures and inadequate sample sizes when the research designs make use of nested structures where multiple participants judge multiple facial stimuli along multiple dimensions.

Despite the limitations presented in this paper, the field of facial morphometrics is a burgeoning field that holds the potential for the development of reliable, inexpensive and non-invasive methods for disease detection and monitoring. For example, the Andreu et al., 2016) is a personal health monitor that integrates a 3D optical scanner, multispectral cameras and gas detection sensor for collecting data of individuals who stand in front of the mirror. One of the key features of the Wize Mirror is the use of facial morphometric analysis to predict cardio-metabolic risk. Studies by Kocabey et al. (2017) and Barr et al. (2018) also reported on the development of Face-to-BMI- Systems that predicts BMI from facial images found on social media platforms, for example. The results reported by these studies indicate that these computer models could predict actual BMI from facial cues alone to a degree of accuracy very similar to that of human observers. With rapid advancement of these types of technologies, exciting possibilities begin to open up for continuous monitoring of health risk associated with BMI.

As mentioned throughout this paper, one of the key conditions that need to be met for facial adiposity to serve as a valid cue to attractiveness and health, is that people need to be able to accurately judge body mass from facial cues. The meta-analysis presented in this review found that participants can reliably estimate BMI from facial cues alone (*r* = 0.71, *n* = 458) and that ethnicity of the rated faces did not mediate the relationship between perceived facial adiposity and estimates of body mass. This is an important finding as it demonstrates that cues related to facial adiposity can be reliably detected by participants and also reliably used to make inferences about another person's body weight. Due to the strong relationship between body weight and negative health outcomes, accurate judgment of facial cues related to body weight, is a key factor in allowing us to make inferences about another person's health.

## Author Contributions

SdJ and VC contributed conception and design of the study; SdJ and VC organized the database; SdJ performed the statistical analysis; SdJ and VC wrote the first draft of the manuscript. All authors contributed to manuscript revision, read and approved the submitted version.

### Conflict of Interest Statement

The authors declare that the research was conducted in the absence of any commercial or financial relationships that could be construed as a potential conflict of interest.
